# Avoiding hot-spots in Microwave-assisted Pd/C catalysed reactions by using the biomass derived solvent γ-Valerolactone

**DOI:** 10.1038/s41598-018-28458-y

**Published:** 2018-07-12

**Authors:** Elena Petricci, Caterina Risi, Francesco Ferlin, Daniela Lanari, Luigi Vaccaro

**Affiliations:** 1 Università degli Studi di Siena, Dipartimento di Biotecnologie, Chimica e Farmacia, Siena, 53100 Italy; 2 Università di Perugia, Laboratory of Green Synthetic Organic Chemistry, Dipartimento di Chimica, Biologia e Biotecnologie, Perugia, 06123 Italy; 3 Università di Perugia, Dipartimento di Scienze Farmaceutiche, Perugia, 06123 Italy

## Abstract

Herein, we report the use of γ-valerolactone as a new biomass-derived reaction medium for microwave assisted organic synthesis. The interaction of this solvent with microwaves and its heating profile under microwave irradiation has been fully characterized for the first time, demonstrating its stability and the applicability in microwave assisted Pd/C catalysed reactions avoiding the arcing phenomena frequently observed in these conditions. The use of γ-valerolactone demonstrated to be compatible with aliphatic and aromatic amines in the hydrogen transfer Pd/C mediated synthesis of benzimidazoles.

## Introduction

The technology used for promoting a synthetic transformation plays a crucial role in the definition of its chemical efficiency and sustainability. In particular, effective heating represents a key challenge for accessing the desired control on the chemical reactivity while ensuring an adequate energy consumption. While flow reactors play certainly a key role for ensuring exceptionally fast heat and mass transfer compared to batch reactors^1^, microwave (MW) dielectric heating still represents nowadays one of the most efficient mean for heating up a reaction mixture and potentially reduce energy consumption^2^ and combine the need for effective synthetic procedures especially on a larger scale^3–6^. The discussion about how MWs are able to accelerate a reaction is still open^7–9^. Even though in most cases the postulated microwaves effects demonstrated to be just related to the erroneous temperature measures^10–15^ or to a misinterpretation of the data obtained^16^, in the last few years the evidence of catalytic MW effects has been observed and demonstrated by different groups especially in reactions involving heterogenous catalysts^8,9,17,18^. Many problems related to reproducibility, predictability and safety issues using MWs have been overcame during the last 30 years, thus transforming MWs in a mature, useful and ordinary technology routinely applied in most organic and medicinal chemistry laboratories as well as in material synthesis and in industrial production^4–6^. Nevertheless, in MW assisted reactions hot spot formation remains a main safety and efficiency problem, responsible for explosions and loss of materials, still remain unsolved right now. Hot spot formation occur with arching phenomena during MW irradiation. They have been widely studied and represent a well documented phenomenon specially occurring because of differential heating of solid catalysts under MW irradiation^9,19–22^. Even though explosions do not occur during the MW irradiation, arching phenomena are responsible for loss of efficiency of the catalytic process especially when charcoal supported metal are used as the catalysts^9,19–22^. The problem has been efficiently overcome in industrial processes by designing dedicated MW reactors^2,19,21^ operating at different frequencies (e.g. 5.8 GHz) than those commonly used in commercially available MW apparata for organic synthesis (2.45 GHz). Alternatively, another solution proposed is to expose the reaction only to magnetic field instead of the electromagnetic one. Nevertheless, it is still not trivial to avoid hot spot formation in traditional MW reactors especially when Pd/C is used as the catalyst in solvents not able to adbsorbe the electromagnetic irradiation (e.g. toluene). Thus hot spot represent one of the main limitations to the use of MW dielectric heating by some organic chemists. It is well documented^20,22,23^ and we directly experienced^24–26^ that hots spots are usually observed when solvents with low boiling points are used in the presence of a heterogeneous catalysts under high electric fields. Sometimes, this issue can be circumvented in lab scale microwave assisted reactions by the use of solvents with higher boiling points than the temperature requested by the process (e.g. dimethylacetamide (DMA), dimethylformamide (DMF), *N*-methylpyrrolidone (NMP), ionic liquids). Nevertheless, the use of these solvents is not always compatible with the desired reaction outcome and operators with not specific training and experience in MW assisted reactions could not be able to avoid explosions of reaction vessels inside the MW reactors with several problems in terms of safety, loss of precious starting materials and, sometimes, damages for the instruments. To the best of our knowledge, the ability to overcome the hot spots generation in MW-assisted reactions using commercially available MW reactors, represents the the last obstacle to overcome in order to spread a secure use of MW heating and consent safer protocols to be defined for both lab scale application in drug discovery and a sustainable industrial production of many different products. On the other hand, it is well known that regulations on the use of highly toxic solvents are now very strict^27,28^. More in general the “solvent issue” in the chemical production is not only related to its suitability for the specific process but also to the waste associated to usage^29^. In fact, solvents, account for a very large contribution of the environmental footprint associated to key production areas such as the pharmaceutical industry^30^. In fact, current interest is therefore focused on the replacement of solvents deriving from fossil resources by selecting new non-toxic alternatives easily accessible from biomass^27,28,31–35^. We have been developing our research to contribute to the development of green synthetic tools by using safer media, heterogenous catalysis and waste-minimized flow protocols^35–41^. Recently, we have focused our attention to γ-valerolactone (GVL)^42–58^, a chemical typically produced by hydrogenation of lignocellulosic biomass-derived levulinic acid^59–63^, possessing very intriguing physical-chemical properties that prompted us to investigate its use in cross-coupling reactions^43–46^ and C–H activation processes^47–52^, to replace traditional polar aprotic solvents.

In the context of using MWs for effective synthesis of natural products and pharmaceutically relevant ingredients^64–67^, we have faced a specific issue in the route towards the definition of an efficient protocol for the MW-assisted, Pd/C catalysed one-pot synthesis of polyfunctionalized benzimidazoles by a hydrogen transfer reaction^25^. The developed protocol takes advantages from MWs and Pd/C interaction, while the reaction does not proceed at all under traditional heating using the same catalyst and conditions. This very efficient, atom economical methodology, able to furnish decorated benzimidazoles in 90 min MW irradiation, resulted to be efficient only in toluene, a solvent not fully adequate for MW assisted reactions^20–23^. The inappropriateness of toluene for Pd/C catalyzed reactions under MW irradiations was confirmed by the frequent hot spot formations and explosions occurred during the optimization of benzimidazole synthesis. In fact, the surface of the activated carbon support of Pd/C catalyst frequently remain dry at the high temperatures (170 °C) requested for this transformation, thus generating arching phenomena (see Fig. S1 and Video S1). More recently, looking for an efficient and sustainable way to overcome these arching phenomena, the use of solvents with higher boiling points has been carefully evaluated with no success. In fact, irradiating with MWs *o*-phenylendiamine (**1a**) and BuNH_2_ (**2a**) in the presence of crotonitrile, AcOH (0.1 eq.) and a 10 mol% of Pd/C, lower conversions were observed at 170 °C using DMF, NMP, or cyclopentyl methyl ether (CPME) compared to toluene (see Table S1). Therefore, we directed our attention towards the non-toxic biomass-derived GVL, that with its 208 °C boiling point, could be a possible green alternative to toluene helping to solve the arching phenomenon formation. To the best of our knowledge the possible use of GVL to overcome the hot spot formation when solid metal catalysts as Pd/C are employed under MW irradiation, it has never been investigated so far and not real solution to the arching phenomena formation exist right now using commercially available MW reactors.

## Results

**Figure 1:**
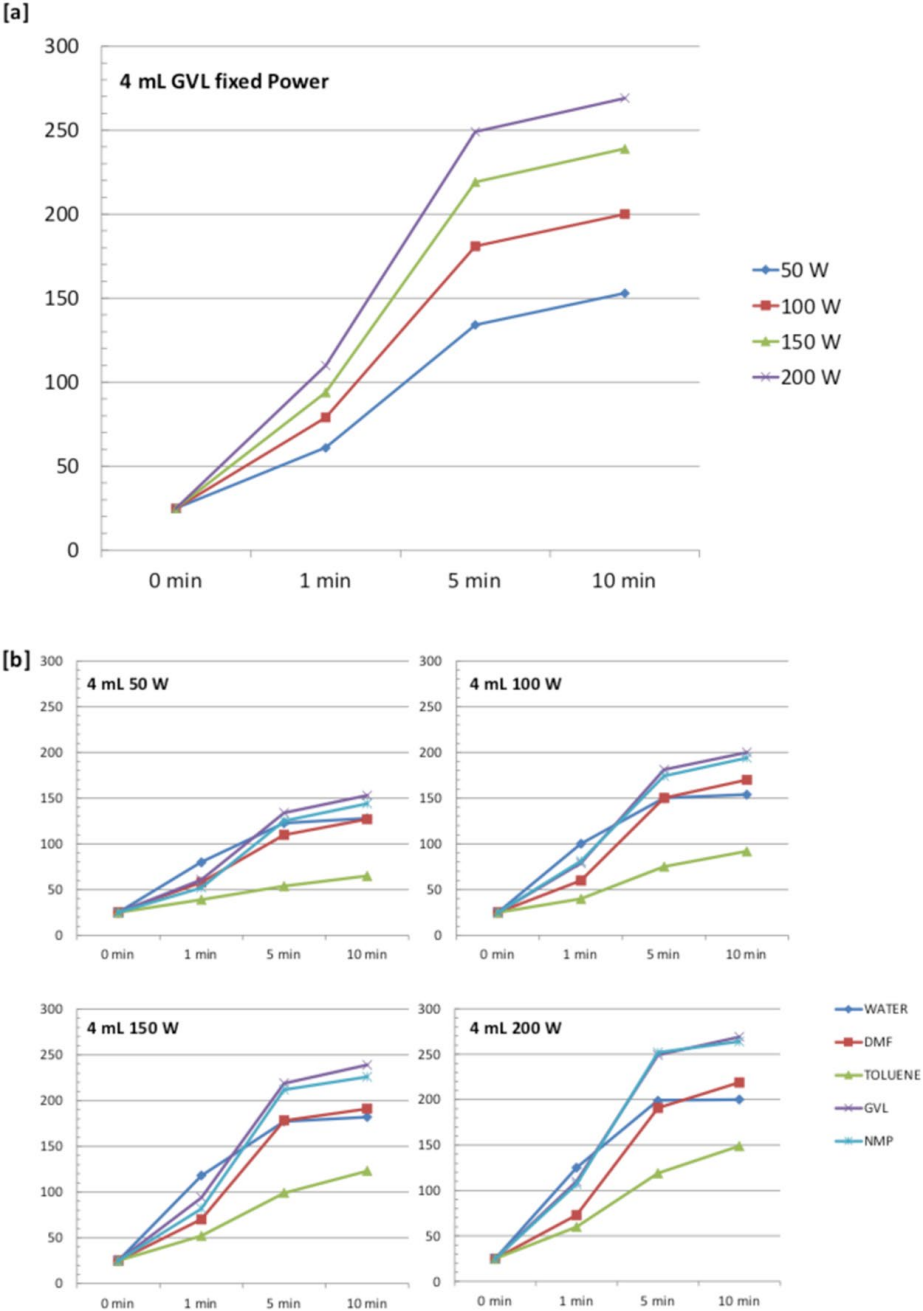
(**a**) Heating profile of GVL under MW irradiation. (**b**) Heating profiles of different solvents under MW irradiation at different fixed powers.

As GVL properties under MW irradiation are completely unexplored, we firstly recorded the heating profiles under MW dielectric heating of GVL to evaluate its adequacy for MW assisted reactions. At this aim, 4 mL of GVL were irradiated for 10 min at 50, 100, 150, and 200 Watt fixed power respectively, and the heating profiles recorded have been summarized in Fig. 1a. It is interesting to highlight that the curves obtained are almost identical for all the four conditions tested while the temperature measured are proportional to the power used (see Table S2). The same MW irradiation conditions were applied to water, DMF, toluene, and NMP for a better characterization of the GVL heating profiles with respect to already characterized solvents (Fig. 1b). GVL interacts with MW in a comparable manner than NMP, the only main difference being represented by the higher final temperature reached with GVL. The biomass-based solvent, demonstrated to be a strongly MWs absorbing compound able to reach very high temperatures in short times, and without the need for the common long ramp times requested for example by toluene or water. It is interesting to note that no degradation of GVL was observed even after irradiation for longer than 10 min at 200 W, while NMP decomposes after 10 min at 150 and/or 200 W.

These data suggest that GVL is a valuable candidate for becoming a really good medium to define safe and efficient MW assisted processes. To demonstrate this thesis, we focused on the benzimidazole synthesis protocol as a representative hydrogen transfer reaction. Infact, this process proved to be very sensitive to the reaction medium and only toluene allowed the proper chemical efficiency to prepare the desired products but leaving room for continuous dangerous formation of hot spot and consequent explosions. This process appers to be ideal for investigating the efficiency in the use of GVL under MWs irradiation including its influence in the hot spot formation.

Nevertheless, as GVL is a lactone, its chemical stability in the presence of an amine^68–70^, which is one the reactant participating the benzimidazole synthesis makes this process even more challenging for GVL and for this reason in our opinion, even more worthy to be investigated.

**Figure 2:**
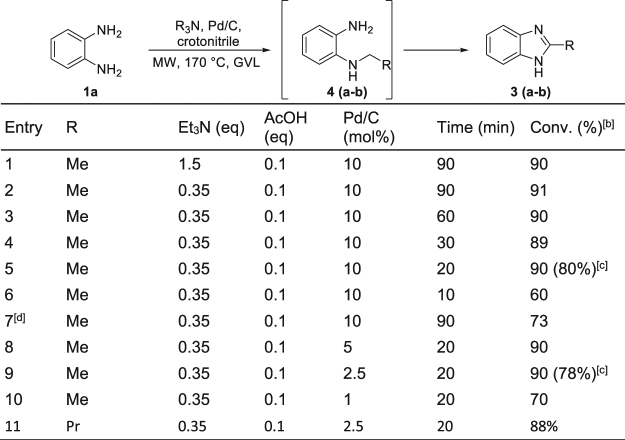
Optimization and use of GVL as medium for the MW-assisted synthesis of benzimidazoles by Pd/C catalysed hydrogen transfer. (**a**) Reaction conditions: **1a** (0.14 mmol), AcOH (10 mol%), 0.5 mL GVL. (**b**) Conversion of **1a** to **3a** determined by GC analysis. (**c**) Isolated yield of the pure product. (**d**) Reaction performed in toluene (1 mL).

Initially, we decided to use triethylamine (1.5 eq.) with the representative substrate *o*-phenylendiamine (**1a**) in GVL in the presence of crotonitrile (2 eq) as the hydrogen acceptor, AcOH (0.1 eq.), 10 mol% of Pd/C, irradiating with MWs at 170 °C for 90 minutes (Fig. 2, entry 1).

**Figure 3:**
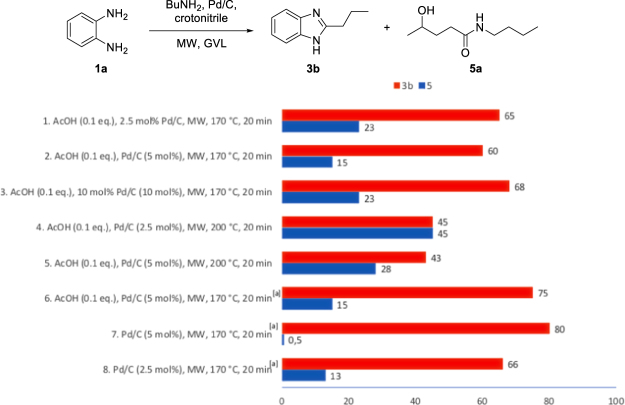
MW-assisted synthesis of benzimidazole **3b** in GVL. (**a**) Dry GVL.

We satisfactorily achieved a 90% conversion to **3a** (measured by GC analysis) without detecting any traces of the intermediate **4a** or of GVL degradation by-products. With this promising data in hand, we have also found that by using 0.35 equivalents of Et_3_N there was no impact on the conversion (Fig. 2, entry 2), proving that two of the three groups of the tertiary amine could be transferred to **1a**^25^. Similar results were obtained irradiating for 60, 30 or even only 20 minutes (Fig. 2, entry 3–5), while irradiating for just 10 minutes in GVL (Fig. 2, entry 6) or using toluene as the solvent for 90 minutes (Fig. 2, entry 7)^25^ lower conversions were observed. Irradiation of **1a** in GVL led to good reaction yields also when the catalyst amount was reduced to 5 or even 2.5 mol% (Fig. 2, entries 8–9), while when 1 mol% was used conversion reached only 70% (Fig. 2, entry 10). The same protocol has been extended to Bu_3_N obtaining also in this case very good conversions and suggesting a more general applicability to tertiary amines (Fig. 2, entry 11). In none of the reactions using GVL, the formation of hot spots was detected, while when toluene was used as medium (Fig. 2, entry 7), the process needed to be repeated 3 times to obtain the reported results and in two out of the three experiments arching phenomena destroyed the reaction tube. Starting from these promising results, we were particularly intrigued about the possibility to study the methodology using primary amines in the presence of GVL. Therefore, **1a** was irradiated in the presence of 1 equivalent of BuNH_2_ (**2a**) in the same reaction conditions reported in the optimized conditions (Fig. 2, entry 9: 2.5 mol % Pd/C, crotonitrile (2 eq.), AcOH (0.1 eq.), MW, 170 °C, 20 minutes). Under these conditions, benzimidazole **3b** was formed in 65% yield accompanied by the presence of 16% of the by-product **5a** resulting from the nuclephilic attack of BuNH_2_ on GVL (Fig. 2). Although this result was expected^68–70^ we further investigated this transformation using higher quantity of Pd/C (5 mol%) at different temperatures always observing an increasing amount of by-product **5a** (Fig. 3). Promising results have been finally obtained using dry GVL (molecular sieves) with the formation of only 15% of **5a** and a 75% conversion into **3b**. The best results have been achieved by irradiating in dry GVL, with 5 mol% of Pd/C, 2 equivs of crotonitrile at 170 °C for 20 minutes in the absence of AcOH. In the previously developed procedure in toluene, AcOH demonstrated to be crucial for an efficient synthesis of benzimidazoles *via* the hydrogen transfer protocol, while in the case of GVL this acid just catalysed the lactone ring opening lowering the conversion to desired product **3b**.

**Figure 4:**
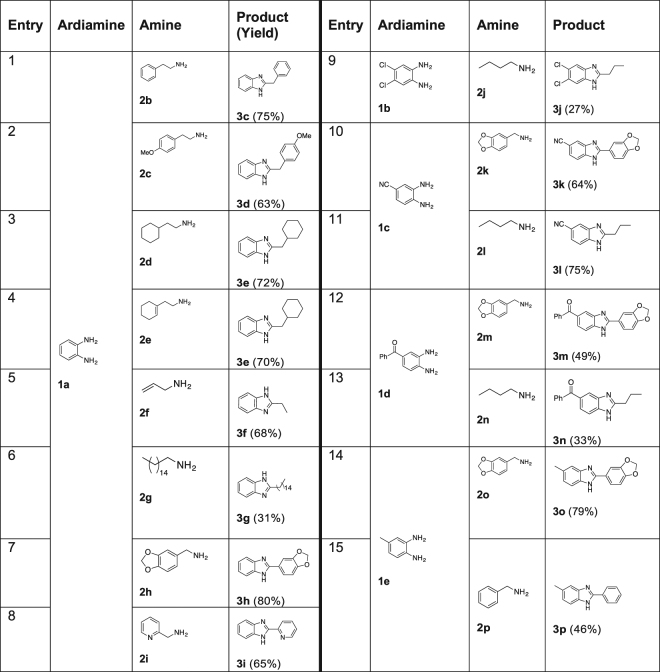
Optimization and use of GVL as medium for the MW-assisted synthesis of benzimidazoles by Pd/C catalysed hydrogen transfer. (**a**) Reaction conditions: arendiamine (0.28 mmol), amine (0.36 mmol), crotonitrile (0.56 mmol), solvent (1 mL), Pd/C (0.014 mmol), MW, 170 °C.

Different *o*-phenylendiamine derivatives and primary amines have been treated in the same reaction conditions and it is interesting to note that good results in term of both conversions and isolated yields are observed using phenethylamine **2b** as well as *p*-methoxyphenethylamine **2c** (Fig. 4, entry 1–2)^25,71^. The same reaction product (**3e**) was obtained using both amines **2d** (Fig. 4, entry 3) and **2e** (Fig. 4, entry 4) thus demonstrating the incompatibility of this protocol with double bonds even using excess of crotonitrile as the hydrogen acceptor^72^. This finding was confirmed by the experiments with allyl amine that furnished the **3 f** in 68% yield (Fig. 4, entry 5).

The reaction yields dramatically lowered down using hexadecan-1-amine (Fig. 4, entry 6) probably because of some solubility problems observed during the transformation, while a great 80% yield is observed with piperonylamine **2 h** (Fig. 4, entry 7). Very good results were obtained using 2-picolylamine obtaining with an almost quantitative conversion, the highly valuable benzimidazole derivative **3i** (Fig. 4, entry 8) used for its luminescent properties in dyes-synthesizer solar cells^73^ and as a metals scavenger because of its ability to complex them. The amine used seems to do not impact in the outcome of the reaction. Otherwise the phenylendiamine derivative used plays a key role in this transformation that furnished lousy results in the presence of halogens (Fig. 4, entry 9).

A Pd mediated dehalogenation of the aromatic ring contemporary with GVL ring opening by the amine used was always observed, indicating that the starting halogenated phenylendiamine derivatives are not nucleophilic enough to react with the intermediate imine formed. Excellent results were observed using 3,4-diaminobenzonitrile **1c** independently on the amine substrate (Fig. 4, entry 10–11), while (3,4-diaminophenyl)(phenyl)methanone (**1d**) and 4-methylbenzene-1,2-diamine (**1e**) furnish only acceptable yields especially with butylamine (Fig. 4, entry 12–15)^74^. The protocol developed consents to obtain benzimidazole derivatives in a more efficient way both in term of reaction yields (**3c**, **3d**, **3h**, **3l**), times (20 min versus 90 min), and safety issues (no hot spots observed using GVL) with respect to the original protocol^25^.

**Figure 5:**
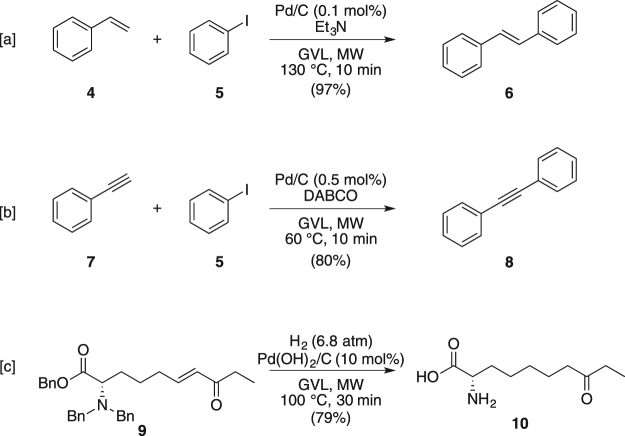
MW-assisted Heck and Sonogashira couplings, and reductions using GVL as the solvent.

The use of GVL for avoiding arching phenomena in Pd/C catalyzed MW assisted transformations was also investigated using Heck^44^ and Sonogashira^45^ cross couplings, and in reduction/hydrogenolysis as model^75^ (Fig. 5). The expected products were always obtained in very good yields without any explosion or hot spot formation observed, thus demonstrating the general applicability of GVL in microwave assisted Pd/C catalysed reactions.

## Methods

All reagents were used as purchased from commercial suppliers without further purification. The reactions were carried out in oven dried or flamed vessels. Solvents were dried and purified by conventional methods^1^ prior use. All MW assisted reactions are performed in a CEM Discover microwave oven equipped with a 10 mL tube for reactions under pressure and an external IR sensor to the detect the reaction temperature during the irradiation (CEM Corporation). Flash column chromatography was performed with Aldrich silica gel 60, 0.040–0.063 mm (230–400 mesh). Aldrich aluminium backed plates pre-coated with silica gel 60 (UV254) were used for analytical thin layer chromatography and were visualized by staining with a KMnO_4_ or Ninidrine solutions. NMR spectra were recorded at 25 °C and 400 or 600 MHz for ^1^H and 100 or 150 MHz for ^13^C. The solvent is specified for each spectrum. Splitting patterns are designated as s, singlet; d, doublet; t, triplet; q, quartet; m, multiplet; br, broad. Chemical shifts (*δ*) are given in ppm relative to the resonance of their respective residual solvent peaks. Low resolution mass spectroscopy analyses were recorded by electrospray ionization.

### General procedure for the determination of heating profiles GVL, toluene, NMP, H_2_O and DMF under MW irradiation

In a 10 mL MW tube, previously dried in oven at 120 °C for 1 h and cooled under N_2_, 4 mL of solvent were added and the solution irradiated with MW for 10 min at 50, 100, 150, and 200 Watt fixed power respectively (set Tmax = 300 °C, set Pmax: 200 PSI).

### General procedure for the synthesis of benzimidazoles

To a solution of the *o*-phenylendiamine (0.28 mmol) in dry GVL (1 mL) triethylamine (0.098 mmol), crotonitrile (46 µL, 0.56 mmol), AcOH (0.028 mmol) and Pd/C 10% wt wet with 50% of water (15 mg, 0.007 mmol) were added. The mixture was irradiated with MWs for 20 min at 170 °C (max internal pressure 200 psi). The crude reaction mixture was filtered and evaporated under reduced pressure. The crude mixture was purified by flash chromatography.

### 2-methyl-1H-benzo[d]imidazole (3a)

^1^H-NMR (400 MHz, CDCl_3_, *δ* ppm): 8.51 (bs, 1H), 7.53 (t, *J* = 3.5 Hz, 2H), 7.21–7.19 (m, 2H), 2.63 (s, 3H). ^13^C-NMR (100 MHz, CDCl_3_, δ ppm): 150.85, 138.19, 121.75, 114.04, 14.48. Anal. Calc. for C_8_H_8_N_2_: C, 72.70; H, 6.10; N, 21.20; found: C, 72.75; H, 6.09; N, 21.22. Eluent: gradient EtOAc to EtOAc:*i*PrOH (90:10). Yield 90%.

### General procedure for the synthesis of benzimidazoles

To a solution of the proper dianiline (0.28 mmol) in dry GVL (1 mL) the proper amine (0.36 mmol), crotonitrile (46 µL, 0.56 mmol) and Pd/C 10% wt wet with 50% of water (30 mg, 0.014 mmol) were added. The mixture was irradiated with MWs for 20 min at 170 °C (max internal pressure 200 psi). The crude reaction mixture was filtered and evaporated under reduced pressure. The crude mixture was purified by flash chromatography.

### 2-propyl-1H-benzo[d]imidazole (3b)

^1^H-NMR (400 MHz, CDCl_3_, δ ppm): 10.84 (bs, 1H), 7.55–7.53 (m, 2H), 7.20–7.18 (m, 2HI, 2.95 (t, *J* = 7.2 Hz, 2H), 1.88 (q, *J* = 7.3 Hz, 2H), 0.95 (t, *J* = 7.1 Hz, 3H). ^13^C-NMR (100 MHz, CDCl_3_, δ ppm): 155.21, 137.95, 121.84, 121.73, 114.11, 30.69, 21.34, 13.41. Anal. Calc. for C_10_H_12_N_2_: C, 74.97; H, 7.55; N, 17.48; found C, 74.99; H, 7.58; N, 17.47. Eluent: gradient EtOAc to EtOAc:*i*PrOH (90:10). Yield 80%.

### 2-benzyl-1*H*-benzo[*d*]imidazole (3c)

^1^H-NMR (400 MHz, CDCl_3_, δ ppm) 8.42 (bs, 1H), 7.46 (bs, 2H), 7.19–7.17 (m, 7H), 4.20 (s, 2H). ^13^C-NMR (100 MHz, CDCl_3_, *δ* ppm): 152.93, 137.02, 135.48, 128.54, 128.49, 126.83, 122.38, 114.24, 34.82. Anal. Calc. for C_14_H_12_N_2_: C, 80.74; H, 5.81; N, 13.45; found: C, 80.80; H, 5.78; N, 13.48. Eluent: gradient EtOAc to EtOAc:*i*PrOH (90:10). Yield 75%.

### 2-(4-methoxybenzyl)-1*H*-benzo[*d*]imidazole (3d)

^1^H-NMR (400 MHz, CDCl_3_, *δ* ppm): 7.50–7.48 (m, 2H), 7.19–7.17 (m, 4H), 6.83 (d, *J* = 8.8 Hz, 2H), 4.20 (s, 2H), 3.75 (s, 3H). ^13^C-NMR (100 MHz, CDCl_3_, *δ* ppm): 158.27, 153.61, 137.97, 134.27, 129.60, 129.15, 127.88.124.56, 121.87, 119.85, 116.34, 114.33, 113.85, 113.52. Anal. Calc. for C_15_H_14_N_2_O: C, 75.61; H, 5.92; N, 11.76; O, 6.7; found: C, 75.64; H, 5.89; N, 11.79. Eluent: gradient EtOAc to EtOAc:*i*PrOH (90:10). Yield 63%.

### 2-(cyclohexylmethyl)-1*H*-benzo[d]imidazole (3e)

^1^H-NMR (400 MHz, CDCl_3_, *δ* ppm): 7.53–7.50 (m, 2H), 7.20–7.17 (m, 2H), 2.76 (d, *J* = 7.2 Hz, 2H), 1.90-1.83 (m, 1H), 1.80–1.53 (m, 4H), 1.15–0.94 (m, 6H). ^13^C-NMR (100 MHz, CDCl_3_, *δ* ppm): 137.84, 128.54, 126.80, 121.94, 121.75, 114.14, 37.35, 36.64, 35.44, 32.76, 25.58, 0.59. Anal. Calc. for C_14_H_18_N_2_: C, 78.46; H, 8.47; N, 13.07; found: C, 78.44; H, 8.49; N, 13.00. Eluent: gradient EtOAc to EtOAc:*i*PrOH (90:10). Yield 75%.

### 2-ethyl-1H-benzo[d]imidazole (3f)

^1^H-NMR (400 MHz, CDCl_3_, *δ* ppm): 8.70 (bs, 1H), 7.63–7.42 (m, 2H), 7.33–7.07 (m, 2H), 2.98 (q, J = 7.6 Hz, 2H), 1.42 (t, J = 7.6 Hz, 3H). ^13^C-NMR (100 MHz, CDCl_3_, *δ* ppm): 155.92, 137.94, 122.32, 122.00, 121.62, 121.27, 114.12, 22.39, 22.16. Anal. Calc. for C_35_H_29_N_7_O_2_: C, 72.52; H, 5.04; N, 16.91; O, 5.52; found: C, 72.54; H, 5.10; N, 16.90. Eluent: gradient EtOAc to EtOAc:*i*PrOH (90:10). Yield 68%.

### 2-hexadecyl-1*H*-benzo[*d*]imidazole (3g)

^1^H-NMR (400 MHz, CDCl_3_, *δ* ppm): 7.52 (dd, *J* = 5.9, 3.2 Hz, 1H), 7.19 (dd, *J* = 6.0, 3.2 Hz, 2H), 2.89 (t, *J* = 7.7 Hz, 2H), 1.83 (p, *J* = 7.7 Hz, 2H), 1.44–1.12 (m, 24H), 0.85 (t, *J* = 6.7 Hz, 3H). ^13^C-NMR (100 MHz, CDCl_3_, *δ* ppm): 134.30, 130.48, 128.40, 121.96, 119.84, 116.32, 65.16, 31.51, 30.16, 29.26, 28.93, 27.79, 22.28, 18.78, 13.71, 13.31. Anal. Calc. for C_23_H_38_N_2_: C, 80.64; H, 11.18; N, 8.18; found: C, 80.65; H, 11.20; N, 8.15. Eluent: gradient EtOAc to EtOAc:*i*PrOH (90:10). Yield 31%.

### 2-(benzo[*d*][1,3]dioxol-5-yl)-1*H*-benzo[*d*]imidazole (3h)

^1^H NMR (400 MHz, *δ* ppm): 7.51–7.46 (m, 4H), 7.14–7.12 (m, 2H), 6.82 (d, J = 8 Hz, 1H), 5.93 (s, 2H). ^13^C-NMR (100 MHz, CDCl_3_, *δ* ppm): 151.20, 148.99, 147.94, 138.40, 129.30, 123.48, 122.41, 121.92, 120.63, 114.49, 108.65, 107.91, 106.74, 101.16. Anal. Calc. for C_14_H_10_N_2_O_2_: C, 70.58; H, 4.23; N, 11.76; O, 13.43; found: C, 70.55; H, 4.21; N, 11.78. Eluent: gradient EtOAc to EtOAc:*i*PrOH (95:5). Yield 80%.

### 2-(pyridin-2-yl)-1*H*-benzo[*d*]imidazole (3i)

^1^H-NMR (400 MHz, CDCl_3_, *δ* ppm): δ 8.61 (d, *J* = 5.0 Hz, 1H), 8.46 (d, *J* = 7.9 Hz, 1H), 7.85 (td, *J* = 7.7, 1.9 Hz, 1H), 7.62 (s, 2H), 7.45 – 7.18 (m, 3H). ^13^C-NMR (100 MHz, CDCl_3_, *δ* ppm): 150.27, 148.63, 147.79, 137.01, 124,0.25, 122.99, 121.48, 115.27. Eluent: gradient EtOAc to EtOAc:*i*PrOH (90:10). Yield 65%^73^.

### 5,6-dichloro-2-propyl-1*H*-benzo[*d*]imidazole (3j)

^1^H-NMR (400 MHz, CDCl_3_, *δ* ppm): 7.62 (s, 2H), 2.87 (t, *J* = 7.6 Hz, 2H), 1.85 (q, *J* = 7.6 Hz, 2H), 1.01 (t, *J* = 7.2 Hz, 3H). ^13^C-NMR (100 MHz, CDCl_3_, *δ* ppm): 156.44, 136.80, 126.18, 115.52, 30.57, 20.95, 13.13. Anal. Calc. for C_10_H_10_Cl_2_N_2_: C, 52.43; H, 4.40; Cl, 30.95; N, 12.23; found: C, 52.45; H, 4.38; N, 12.21. Eluent: gradient EtOAc to EtOAc:*i*PrOH (90:10). Yield 27%.

### 2-(benzo[*d*][1,3]dioxol-5-yl)-1*H*-benzo[*d*]imidazole-5-carbonitrile (3k)

^1^H-NMR (400 MHz, MeOD, *δ* ppm): 7.86 (s, 1H), 7.62–7.56 (m, 3H), 6.93 (d, *J* = 8, 1H), 7.47 (s, 1H). ^13^C-NMR (100 MHz, MeOH, *δ* ppm): 154.56, 149.88, 148.17, 140.56, 138.61, 125.34, 123.49, 122.07, 121.20, 118.92, 117.38, 113.70, 107.97, 106.19, 101.52. Anal. Calc. for C_15_H_9_N_3_O_2_: C, 68.44; H, 3.45; N, 15.96; O, 12.15; found: C, 68.45; H, 3.49; N, 15.97. Eluent: gradient EtOAc to EtOAc:*i*PrOH (90:10). Yield 64%.

### 2-propyl-1*H*-benzo[*d*]imidazole-5-carbonitrile (3l)

^1^H-NMR (400 MHz, CDCl_3_, *δ* ppm): 7.91 (s, 1H), 7.62 (d, *J* = 8 Hz, 1H), 7.51 (d, *J* = 8 Hz, 1H), 2.97-2.93 (m, 2H), 1.94-1.89 (m, 2H), *δ* = 1.03 (t, *J* = 7.2 Hz, 3H). ^13^C-NMR (100 MHz, CDCl_3_, *δ* ppm): 157.52, 140.03, 137.60, 125.81, 119.33, 114.76, 105.17, 30.63, 20.90, 13.38. Anal. Calc. for C_11_H_11_N_3_: C, 71.33; H, 5.99; N, 22.69; found: C, 71.34; H, 6.01; N, 22.71. Eluent: gradient EtOAc to EtOAc:*i*PrOH (90:10). Yield 75%.

### (2-(benzo[*d*][1,3]dioxol-5-yl)-1*H*-benzo[*d*]imidazol-5-yl)(phenyl)methanone (3m)

^1^H-NMR (400 MHz, CDCl_3_, *δ*): 7.96 (d, *J* = 1.6 Hz, 1H), 7.83–7.29 (m, 9H), 6.82 (d, *J* = 8.1 Hz, 1H), 5.94 (d, *J* = 1.1 Hz, 2H). ^13^C-NMR (100 MHz, CDCl_3_, *δ* ppm) 201.00, 157.81, 153.38, 151.87, 146.02, 141.70, 141.46, 135.67, 135.43, 133.45, 131.67, 128.86, 126.42 125.26, 120. 91, 118.10, 112.27, 110.60, 105.18. Anal. Calc. for C_21_H_14_N_2_O_3_: C, 73.68; H, 4.12; N, 8.18; O, 14.02; found: C, 73.65; H, 4.09; N, 8.17. Eluent: gradient EtOAc to EtOAc:*i*PrOH (90:10). Yield 49%.

### Phenyl(2-propyl-1*H*-benzo[*d*]imidazol-5-yl)methanone (3n)

^1^H-NMR (400 MHz, CDCl_3_, *δ* ppm): 8.04 (s, 1H), 7.79–7.70 (m, 3H), 7.61-7.54 (m, 2H), 7.52–7.43 (m, 2 H), 2.94 (t, *J* = 7.2 Hz, 2H), 1.91 (q, *J* = 7.2 Hz, 2H), 1.03 (t, *J* = 7.2 Hz, 3H). ^13^C-NMR (100 MHz, CDCl_3_, *δ* ppm) = 196.75, 156.96, 137.79, 136.80, 131.85, 131.71, 129.57, 127.80, 124.79, 117.19, 113.98, 30.65, 20.95, 13.39. Anal. Calc. for C_17_H_16_N_2_O: C, 77.25; H, 6.10; N, 10.60; O, 6.05; found: C, 77.23; H, 6.15; N, 10.67 Eluent: gradient EtOAc to EtOAc:*i*PrOH (90:10). Yield 33%.

### 2-(benzo[*d*][1,3]dioxol-5-yl)-5-methyl-1*H*-benzo[*d*]imidazole (3o)

^1^H-NMR (400 MHz, CDCl_3_, *δ*) 8.48 (bs, 1H), 7.56–7.19 (m, 4H), 7.01 (d, *J* = 8.2Hz, 1H), 6.71 (d, *J* = 8.1Hz, 1H), 5.89 (s, 2H), 2.39 (s, 3 H). ^13^C-NMR (100 MHz, CDCl_3_, *δ* ppm) 151.42, 148.76, 147.82, 138.33, 137.23, 132.23, 123.84, 123.67, 120.79, 114.49, 113.88, 108.22, 106.79, 101.05, 21.23. Anal. Calc. for C_15_H_12_N_2_O_2_: C, 71.42; H, 4.79; N, 11.10; O, 12.68; found: C, 71.48; H, 4.82; N, 11.09. Eluent: gradient EtOAc to EtOAc:*i*PrOH (90:10). Yield 79%.

### 5-methyl-2-phenyl-1*H*-benzo[*d*]imidazole (3p)

^1^H-NMR (400 MHz, CDCl_3_/CD_3_OD *δ*) = 8.03 (d, *J* = 5.2 Hz, 2H), 7.51(d, *J* = 8 Hz, 1H), 7.40–7.38 (m, 4H), 7.07 (d, *J* = 7.6 Hz, 1H), 2.44 (s, 3 H). ^13^C-NMR (100 MHz, CDCl_3_/CD_3_OD, *δ* ppm) 151.08, 138.196, 136.93, 132.27, 129.47, 128.47, 126.09, 123.89, 114, 44, 113.88, 21.12. Anal. Calc. for C_14_H_12_N_2_: C, 80.74; H, 5.81; N, 13.45; found: C, 80.78; H, 5.79; N, 13.41. Eluent: gradient EtOAc to EtOAc:*i*PrOH (90:10). Yield 46%.

### (E)-1,2-diphenylethene (6)

To a solution of the iodobenzene (57 μL, 0.5 mmol), styrene (69 μL, 0.6 mmol) and triethylamine (69 μL, 0.5 mmol) in dry GVL (0.5 mL), Pd/C 10% wt wet with 50% of water (1 mg, 0.0005 mmol) was added. The mixture was irradiated with MWs for 10 min at 130 °C (max internal pressure 200 psi). The crude reaction mixture was filtered off and the solution diluted with cyclopentyl methyl ether (3 mL) and the organic phase was washed with H_2_O (3 × 2 mL). After evaporation under reduced pressure 6 was obtained as a with solid (87 mg) in 97% yield. M.p.: 123–125 °C. ^1^H-NMR (400 MHz, CDCl_3_ *δ*): 7.54 (d, 4H, *J* = 7.4 Hz), 7.37 (t, 4H, *J* = 8.1 Hz), 7.28 (d, 2 H, *J* = 7.8 Hz), 7.15 (s, 2H). ^13^C-NMR (100 MHz, CDCl_3_, *δ* ppm): 137.52, 129.02, 127.89, 125.71^44^.

### 1,2-diphenylethyne (8)

To a solution of iodobenzene (57 μL, 0.5 mmol), and ethynylbenzene (84 μL, 0.75 mmol) DABCO (70 mg, 0.6 mmol) in GVL (1 mL), Pd/C 10 wt% (5.3 mg, 0.0025 mmol) was added. The mixture was irradiated with MWs for 10 min at 60 °C (max internal pressure 200 psi). Petroleum ether was added and the reaction mixture was filtered over celite pad washing with water and 1 M HCl. The organic layer was dried over dry Na_2_SO_4_ and the solvent was removed under reduced pressure. The crude oil was purified by flash chromatography (petroleum ether) to obtain **8** as a white solid (71 mg) in 80% yield. M.p.: 59–62 °C. ^1^H-NMR (400 MHz, CDCl_3_ *δ*): 7.39–7.44 (m, 6H), 7.61–7.67 (m, 4H). ^13^C-NMR (100 MHz, CDCl_3_, *δ* ppm): 131.82, 129.23, 129.03, 123.54, 89.65^45^.

### (S)-2-amino-8-oxodecanoic acid (10)

To a solution of **9** (100 mg, 0.16 mmol) in dry GVL (1 mL) Pd(OH)_2_/C 10%wt (22 mg, 0.016 mol) was added. The 10 mL vial was introduced into the Discover Microwave Synthesizer and purged three times with vacuum/H_2_ and finally charged with H_2_ (6.8 atm). The mixture was irradiated with MWs microwave irradiation for 30 min at 100 °C (max internal pressure 200 psi). The vial was vented, flushed with nitrogen, and the reaction mixture was filtered over celite pad washing with MeOH. The solvent was evaporated and the cure mixture purified by flash chromatography using AcOEt:MeOH (9:1) obtaining 49 mg of **10** (75% yield). ^1^H-NMR (400 MHz, CDCl_3_/DMSOd_6_ *δ*): 9.00 (bs, 3H), 3.62 (m, 1H), 2.42 (q, 2H, *J* = 7.2 Hz), 2.37 (m, 2H), 1.33–1.90 (m, 8H), 1.15 (t, 3H, *J* = 7.2 Hz). ^13^C-NMR (100 MHz, CDCl_3_/DMSOd_6_ *δ*): δ = 214.18, 175.52, 55.99, 48.66, 29.69, 28.99, 25.91, 24.21, 22.22, 9.85. MS (ES) m/z 202 [M + H]^+^. Anal. Calc. for C_10_H_19_NO_3_ C, 59.68; H, 9.52; N, 6.96; found: C, 59.85; H, 9.55; N, 7.01.

## Electronic supplementary material

Supplementary images

Toluene MW-irradition

GVL MW-irradition
