# Supplementary material for: Avoiding hot-spots in Microwave-assisted Pd/C catalysed reactions by using the biomass derived solvent γ-Valerolactone
Source: Sci Rep. 2018 Jul 12;8:10571. doi: 10.1038/s41598-018-28458-y (PMC6043498; doi:10.1038/s41598-018-28458-y)
Supplement: Supplementary file 1 — Supplementary images [file 41598_2018_28458_MOESM1_ESM.pdf]

Avoiding hot-spots in Microwave-assisted Pd/C catalysed reactions by using the biomass derived solvent γ-Valerolactone

Elena Petricci[1,*], Caterina Risi[1], Francesco Ferlin[2], Daniela Lanari[3], and Luigi Vaccaro[2,*]

[1]Università degli Studi di Siena, Dipartimento di Biotecnologie, Chimica e Farmacia, Siena, 53100, Italy.
[2]Università di Perugia, Laboratory of Green Synthetic Organic Chemistry, Dipartimento di Chimica, Biologia e Biotecnologie, Perugia, 06123, Italy.
[3]Università di Perugia, Dipartimento di Scienze Farmaceutiche, Perugia, 06123, Italy.

[*]elena.petricci@unisi.it; luigi.vaccaro@unipg.it

Supporting Information

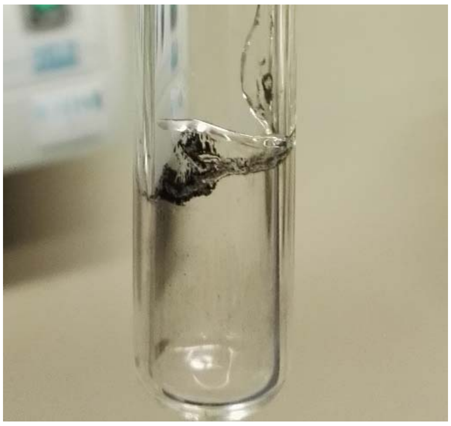

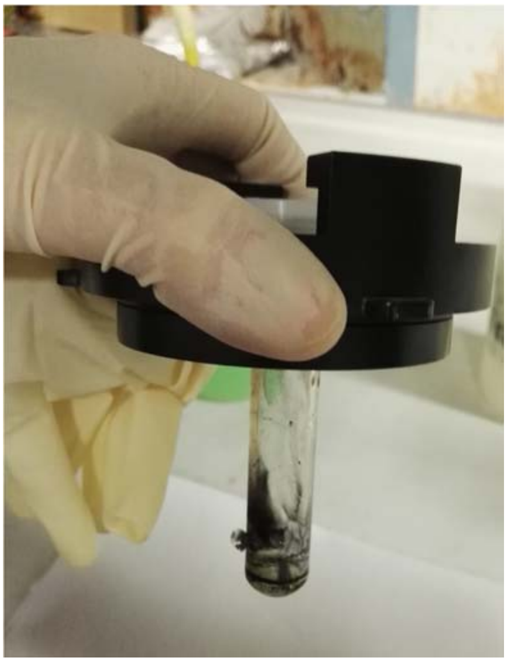

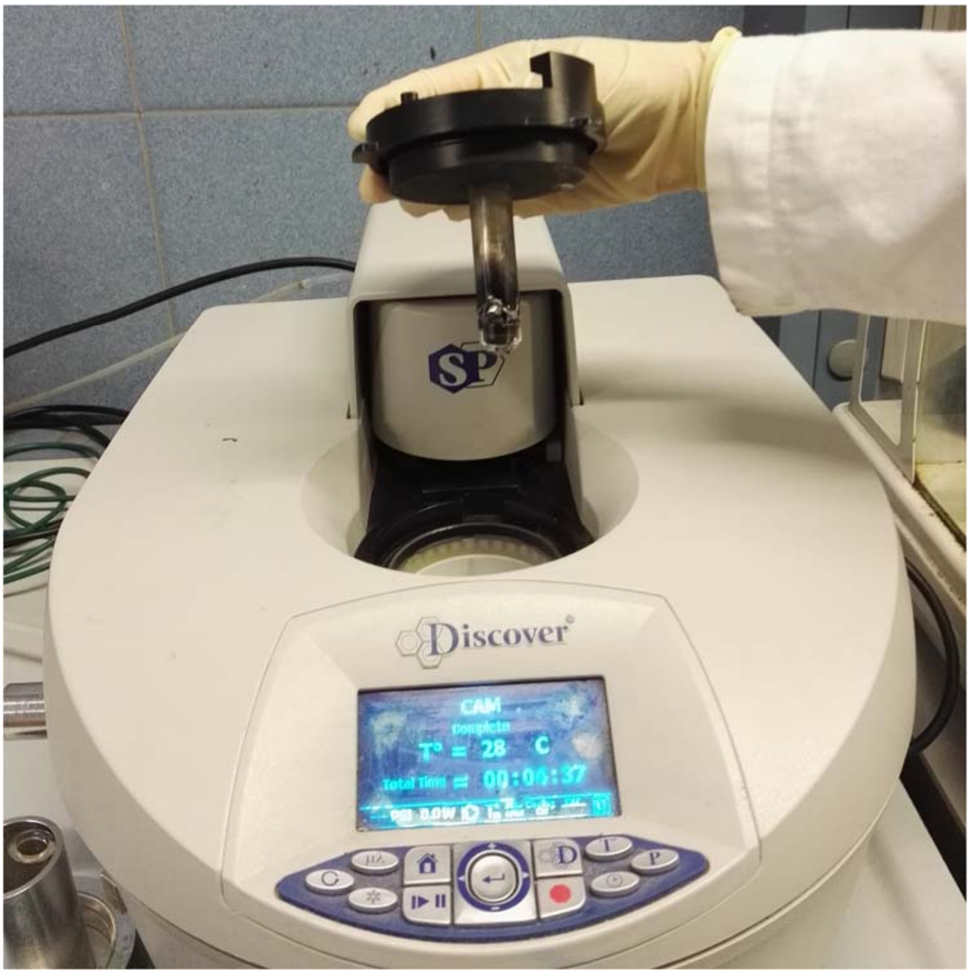

SP
Discover
CAM
Complete
T° = 28 C
Total Time =

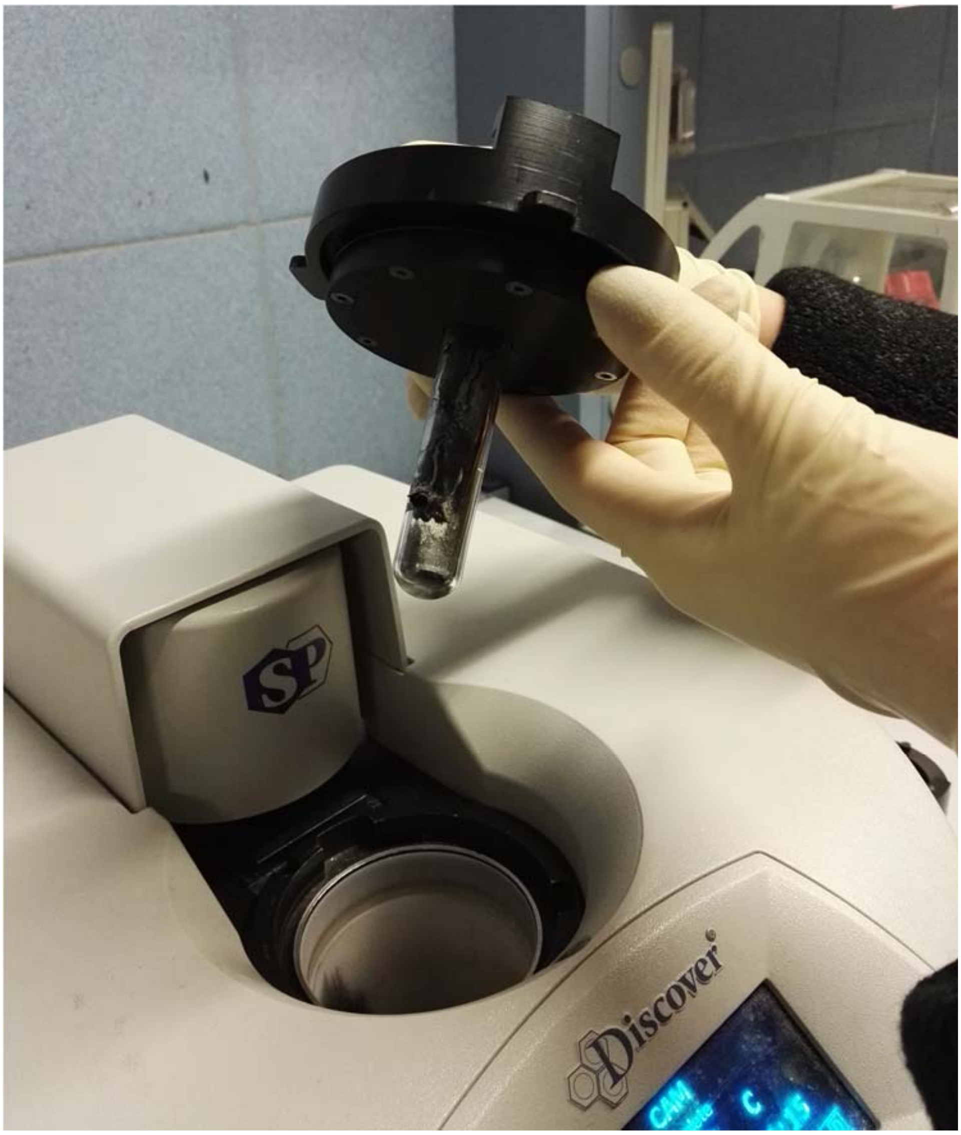

Figure S1. Examples of MW tubes after hot spot formation

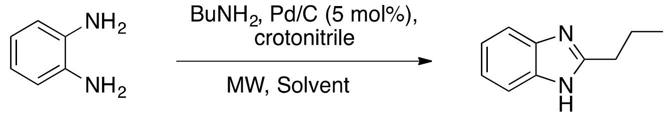

1

3

Entry	Medium	Conv. (%)[b]
1[a]	Toluene	50%
2[a,b]	CPME	20%
3[a,c]	EtOH	18%
4[d]	NMP	81%
5[d]	GVL	89%

Table S1. Synthesis of benzimidazole in different solvents. [a] Reaction conditions: *o*-phenylendiamine (1) (0.28 mmol), $BuNH_2$ (0.36 mmol), crotonitrile (0.57 mmol), solvent (1.6 mL), MW, 170 °C, 20 min; [b.] Maxim temperature reached 140 °C; [c.] Maxim

temperature reached 120 °C. d. Conditions: *o*-phenylendiamine (0.28 mmol), buthylamine (0.36 mmol), crotonitrile (0.57 mmol), solvent (0.8 mL), MW, 170 °C, 20 min.

Power	T (1 min)	T (5 min)	T (10 min)
50 W	61 °C	134 °C	153 °C
100 W	79 °C	181 °C	200 °C
150 W	94 °C	219 °C	239 °C
200 W	110 °C	249 °C	269 °C

Table S2. Recorded temperature during the MW irradiation of 4 mL of GVL at fixed 50, 100, 150 and 200 W.
